# DCS: Distributed Caching Strategy at the Edge of Vehicular Sensor Networks in Information-Centric Networking

**DOI:** 10.3390/s19204407

**Published:** 2019-10-11

**Authors:** Yahui Meng, Muhammad Ali Naeem, Rashid Ali, Yousaf Bin Zikria, Sung Won Kim

**Affiliations:** 1 School of Science, Guangdong University of Petrochemical Technology, Maoming 525000, China; mengyahui@gdupt.edu.cn (Y.M.); malinaeem7@gmail.com (M.A.N.); 2 School of Intelligent Mechatronics Engineering, Sejong University, Seoul 05006, Korea; rashidali@sejong.ac.kr; 3 Department of Information and Communication Engineering, Yeungnam University, Daegu 38541, Korea

**Keywords:** information-centric networking (ICN), client-cache (CC), video on demand (VoD), vehicular sensor network (VSN)

## Abstract

Information dissemination in current Vehicular Sensor Networks (VSN) depends on the physical location in which similar data is transmitted multiple times across the network. This data replication has led to several problems, among which resource consumption (memory), stretch, and communication latency due to the lake of data availability are the most crucial. Information-Centric Networking (ICN) provides an enhanced version of the internet that is capable of resolving such issues efficiently. ICN is the new internet paradigm that supports innovative communication systems with location-independent data dissemination. The emergence of ICN with VSNs can handle the massive amount of data generated from heterogeneous mobile sensors in surrounding smart environments. The ICN paradigm offers an in-network cache, which is the most effective means to reduce the number of complications of the receiver-driven content retrieval process. However, due to the non-linearity of the Quality-of-Experience (QoE) in VSN systems, efficient content management within the context of ICN is needed. For this purpose, this paper implements a new distributed caching strategy (DCS) at the edge of the network in VSN environments to reduce the number of overall data dissemination problems. The proposed DCS mechanism is studied comparatively against existing caching strategies to check its performance in terms of memory consumption, path stretch ratio, cache hit ratio, and content eviction ratio. Extensive simulation results have shown that the proposed strategy outperforms these benchmark caching strategies.

## 1. Introduction

The increasing demands for novel applications due to advancements in technology have led to increased interest in finding a means by which to deliver popular data contents to remote physical locations such as in Vehicular Sensor Networks (VSNs), mainly for Vehicular Ad Hoc Networks (VANET) [1]. In VSNs, vehicles are equipped with diverse onboard units (sensors) for the communication of information. The exponentially-increasing usage of the internet has posed problems for current VSNs due to its need for diverse facilities such as the dissemination of immense amounts of data from heterogeneous consumers along with periodic connectivity in harsh signal propagation, spare roadside conditions, and high levels of mobility [2,3]. It is difficult to provide these facilities to vehicular networks using the IP-based protocols of the present, host-centric connectivity network paradigm [2,3]. However, the Information-Centric Internet (ICN) has proposed emerging technologies to provide novel applications to fulfill future internet requirements. In recent years, the ICN has received significant interest from the research community because of its rapid growth and flexible nature vis-a-vis data communication services. It delivers a unique computing environment in which the router turns into a server. As a result, these servers can modify, understand, and measure the surrounding environment for data dissemination [4]. The immense growth of today’s internet traffic requires high-quality communication services because of network congestion, which has been increasing exponentially [5]. Therefore, the internet is currently facing several problems related to network traffic. For example, the internet requires extra content retrieval latency along with high bandwidth consumption during data dissemination.

Moreover, the usage of resources and energy have also increased. Connected devices are resource-constrained, and connected devices have a significant impact upon communication in everyday life. The basic concept behind ICN technology is that all objects have to operate through processing, identifying, and caching abilities to communicate within a diverse environment and achieve good data dissemination performance [6]. The reason is that the current internet supports an outdated, location-based paradigm in which all devices need to connect through IP addresses that indicates their location. As a result, the IP-based internet is facing several issues, such as communication latency, data searching overhead due to high network congestion, and the dissemination of identical contents many times from remote servers [7,8]. Moreover, the IP-based internet architecture is insufficient to achieve better results in data communication through a large number of devices because location-based communication needs a high amount of energy; this is a fundamental limitation of internet architecture. New research and big data technology will deliver an enormous amount of data that will be challenging for the current, IP-based internet architecture [8,9].

ICN-based projects were designed combining several modules, such as caching, naming, forwarding, mobility, and security. However, caching is the first module that pays full attention to determining the ICN from the IP-based internet architecture. It delivers many benefits during data dissemination such as short stretch, and provides fast data delivery services [10]. ICN focuses on data delivery without location dependency. Thus, this approach makes the ICN architecture beneficial for the internet environment. ICN does not require IP addresses for data dissemination between sources and consumers; rather, it uses unique names to send and retrieve data contents [11]. The cache is the most significant feature of the ICN; it is used to store the transmitted contents near the desired consumers. In vehicular networks, vehicles obtain their required contents from neighboring vehicles in short time periods with small stretch. Therefore, there is no need to forward the coming interests to remote providers, and a large number of user requests can be served locally.

**Figure 1:**
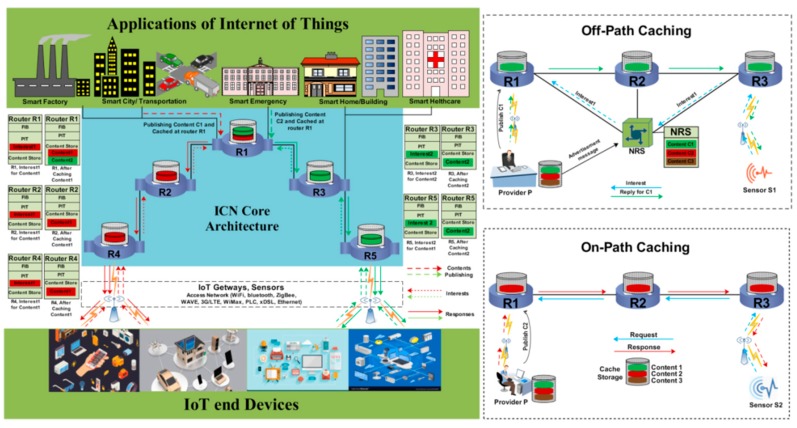
Caching architecture, On-path and Off-path caching.

In the ICN, consumers send their interests directly to the network, and the whole system is responsible for sending the corresponding data to the appropriate consumer. A copy of the disseminated content is cached at different locations between consumers and providers, according to the selected caching strategy. This makes it possible to store the contents in a location which is geographically close to the consumers [12]; therefore, it can reduce latency by caching contents near consumers, because subsequent interests will be satisfied with the cached content. The purpose of the implementation of the in-network cache is to enhance data transmission services and reduce the high amount of network traffic that causes link congestion and increases bandwidth consumption [13]. Moreover, in-network caching can reduce energy and resource consumption because, in the ICN, subsequent interests do not require traversing towards remote servers [14]. ICN caching is divided into two categories, i.e., off-path and on-path caching. In off-path caching a particular entity named Name Resolution System (NRS) is used to broadcast the published contents’ names with their locations. Initially, all the consumer’s interests are transmitted to the NRS, and the NSR forwards these interests to the appropriate data sources, as shown in Figure 1 (Off-Path Caching). In on-path caching, consumers are directly sending their Interests to the network, and the network directly sends back the corresponding contents to the consumer, as illustrated in Figure 1 (On-Path caching). Therefore, it can reduce the communication and computation overhead in data dissemination [15].

In contrast to the ICN cache strategy, there are three exceptional features to take into account when applying ICN cache to VSNs. First, in view of their protection and selfishness, drivers of vehicles may play a tentative role in terms of obeying the guidelines of a cache-sharing strategy [2]. Furthermore, vehicles’ frequent and dynamic topology changes increase the unpredictability of the cache strategy [16]. In addition, vehicles have weak computational and storage resources compared to conventional network base stations (such as access points) and routers, and the cache redundancy of the strategy ought to be diminished [17].

Most of the work done by researchers in this domain has not taken into account and explored the characteristics of VSNs. A vehicle-to-infrastructure scenario cache policy in VSNs is proposed in [18]. The authors proposed an Integer Linear Programming (ILP) definition of the issue of optimally appropriating the contents in the VSN while thinking about the accessible storage limit and connection ability to expand the likelihood that a vehicle will be able to retrieve the desired content. However, due to weak wireless links and mobility, vehicles cannot directly access servers or access points (APs). Therefore, a VSN cache strategy is needed at the edge of the network. For this purpose, this paper implements a new distributed caching strategy (DCS) at the edge of the network in VSN environments to reduce the number of data dissemination problems. The proposed DCS mechanism is studied comparatively against existing caching strategies to check the performance in terms of memory consumption, path stretch ratio, cache hit ratio, and content eviction ratio.

Section 2 provides an overview of related studies. Section 3 defines the problems that still exist in associated studies. In Section 4, the proposed model is explained. In Section 5, the performance evaluation of related and proposed research is done using a simulation platform. In Section 6, the paper is concluded. Finally, Section 7 presents some future directions for Vehicular Sensor Networks.

## 2. Related Study

ICN is an emerging environment in which devices have the ability to respond to their surroundings with the help of caching [19]. Data dissemination is the most fundamental phenomenon of all internet architectures, in which the current IP addresses-based internet is supported by the old version of the architecture for data transmission between remote locations. Therefore, data is distributed when a consumer’s interest is received [20]. The reason for this is that the IP-based internet architecture supports location-based data dissemination that produces serious issues for future communication processes due to the exponential increase in the amount of data traffic. At the same time, ICN delivers location-independent data dissemination and offers lots of benefits in terms of improving the overall data communication process [21]. Therefore, ICN can reduce the critical issues of the IP-based architecture, and can fulfill future internet requirements.

### 2.1. Client-Cache (CC)

In Client-Cache Strategy (CC), the validity of cached contents is observed. The concept of CC is derived from central-based caching, in which the content is cached at routers that are linked to more routers [22]. The aim of CC is to increase the validity of a given content. The validity is measured according to the lifespan of the cached content at intermediate routers and from the publisher. The content is selected as valid if its lifespan of at the publisher is higher than its lifespan which has been cached at an intermediate router.

**Figure 2:**
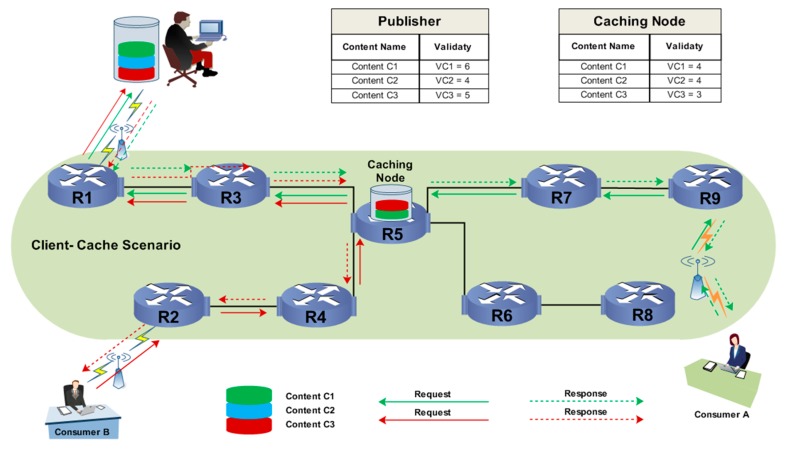
Client-Cache.

In Figure 2 (Client-Cache scenario), various interests from Consumers A and B are sent to retrieve the Content C1. Primarily, the lifespan of Content C1, Content C2, and Content C3 are shown by VC6, VC4, and VC5, respectively, in Figure 2. In CC, the lifespan of the content is taken as VC, which shows the validity of the content. Therefore, the lifespans of contents C1 and C2 are higher at the publisher than at router R5. This indicates that contents C1 and C2 should be cached at router R5; thus, C1 will be cached at router R5, as shown in Figure 2.

### 2.2. Flexible Popularity-Based Caching Strategy (FlexPop)

The FlexPop caching strategy compiles two mechanisms to complete its content caching procedure [23]. Primarily, it performs a content caching procedure to cache transmitted content alongside the data routing path. It executes a second content eviction procedure if the disseminated content does not identify the free cache space for accommodation at the intermediate routers. FlexPop requires the maintenance of a popularity table that helps to count the number of interests at each router for all content names. On the basis of the received interests, the popularity of a given piece of content is calculated in the PT using the content counter and popularity tag. Initially, the content is stored in the PT to calculate its popularity. If the content within the PT indicates that its popularity is equal or greater than the threshold, it forwards it to the comparison table (CT). The CT is responsible for maintaining information about the popular content. It compares the popularity of the new content with the popularities of the previous popular content; if the new content demonstrates more significant demand than the other content, it is labeled as popular, and the CT is shared with the neighboring routers. When the popularity of that content reaches a threshold, the content is forwarded to the router that has the maximum number of outgoing interfaces to be cached. If the cache of the router having the maximum outgoing interfaces is overflowing, the content is recommended for caching at the router that is associated with the second-highest number of outgoing interfaces.

**Figure 3:**
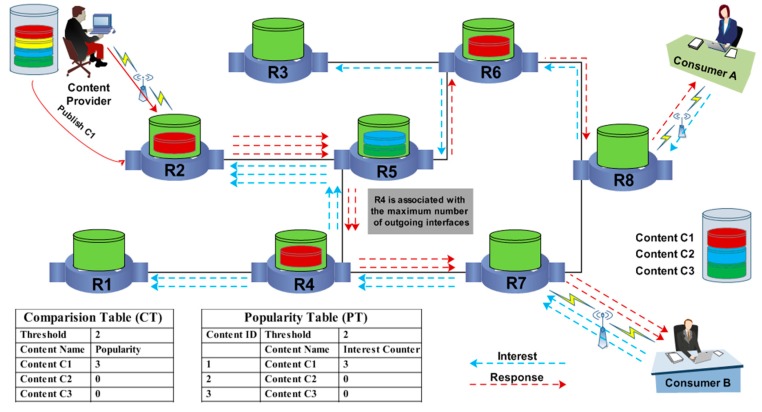
Flexible Popularity-based Caching Strategy.

Figure 3 illustrates the content caching procedure in FlexPop. Initially, two contents, C2 and C3, are cached at router R5. Router R5 is associated with the maximum outgoing interfaces, and only two pieces of content can reside in its cache owing to its limited capacity. Three interests from consumers A and B are sent to router R2 to retrieve content C2. In response to the received interests, the router R2 becomes the provider and sends content C1 to consumers A and B. At the same time, the popularity of content C1 is measured on the basis of the received interests for content C1. According to FlexPop, C1 gains the highest popularity, as shown by the CT in Figure 3; therefore, it is labeled “popular” and recommended for caching at the router with the maximum number of outgoing interfaces (i.e., router R5). However, there is no free space at router R5 for caching content C1; therefore, it will be cached at the router having the second-highest number of outgoing interfaces. Thus, C1 will be cached at routers R4 and R6.

### 2.3. Centrality-Based Caching Strategy (CCS)

**Figure 4:**
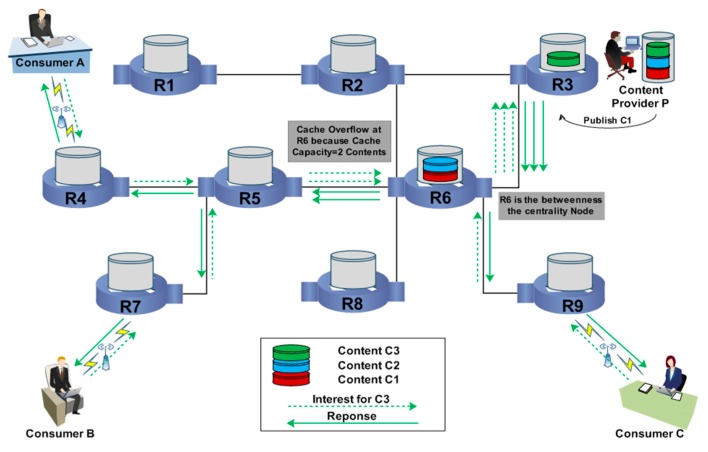
Centrality-Based Caching Strategy.

This content caching mechanism requires two approaches. First, it determines the betweenness centrality node by calculating the links associated with each node. Second, it decides how to cache the transmitted content along the data routing path [24]. In this caching mechanism, the interesting content is forwarded to the node that has the maximum number of outgoing interfaces or the maximum number of paths associated with it. If a node is associated with a high number of data routing paths, it has more opportunities to cache the disseminated content [25]. Figure 4 illustrates the content caching mechanism using centrality-based caching in which Consumers A, B, and C are associated with routers R4, R7, and R9, respectively. These consumers sent three interests to retrieve content C1, as that content is already published in the network by the content provider (P). As the interests for content C1 reach router R3, the required content is obtained. Therefore, router R3 acts as a provider and transmits content C1 to the interested consumers (i.e., A, B, and C). During the transmission of the content, each router calculates the number of data routing paths associated with it. According to the caching nature of the CCS, router R6 is selected as the betweenness centrality router because it has the highest number of paths associated with it along the data delivery path between the provider and the consumers. Hence, content C1 will be cached at R5.

## 3. The Problem Description

ICN provides centrality-based caching strategies in which the transmitted content is cached at a betweenness centrality location to fulfill the requirements of subsequent interests [26]. However, these caching strategies have been facing some critical issues due to the limited capacity of cache storage at the betweenness centrality location.

The CC tries to improve content validity, but also introduced some problems such as content eviction ratio and the stretch ratio between the consumer and the provider, because the content’s legality must be measured at all the routers, which takes time. According to CC, the interesting content will be cached at a betweenness centrality router that increases the number of significant issues which occur due to caching the transmitted content only at one router, such as memory consumption. Moreover, it increases the path length due to the high content eviction rate between the consumer and the provider. The reason for this is that all the interests need to be forwarded to the primary publisher due to the limited cache capacity at the betweenness centrality location. Another issue of CC is that if a large number of interests are received for Content C, and the validity of C is the same at the betweenness centrality node and the server, then according to the CC, Content C will not cache at the betweenness centrality router even, it is deemed to be popular. Therefore, all the interests for popular content will be forwarded to the main server that maximizes the stretch, and the cache hit ratio will automatically be decreased. The amount of cache storage is limited, and it is difficult to accommodate all the content at the betweenness centrality router. Therefore, certain problems arise in CCS that demonstrate the increased congestion which can occur at the centrality position, leading to a high number of evictions within short intervals of time. The reason for this is that if the cache of the betweenness centrality position becomes full, all the interest for content must to be forwarded to the remote provider. In addition, it does not care about the content popularities, which increases the caching of contents with lower popularities. Thus, the overall cache hit ratio decreases because several interests have to be accomplished from remote providers owing to the large accommodation of less popular content. Hence, the overall caching performance is decreased [27].

FlexPop was developed to solve important problems such as high memory consumption, high evictions, and stretch. However, it increases content homogeneity through multiple replications of the same content. Consequently, it retains the process of content evictions and higher resource utilization. Moreover, there is no criterion by which to choose popular content according to time consumption. We assumed a case where three interests were generated for content C1 in 5 s, and two for content C2 in 1 s. According to FlexPop, C1 will be the most popular because no time distinction is included for the selection of popular content. Consequently, the most recently-used content will remain unpopular, which causes a low cache hit ratio that affects the efficiency of the content dissemination and increases the content eviction ratio. Moreover, in FlexPop, two tables, PT and CT, must be computed for each piece of content and to identify popular content, which increases the searching overhead during the selection of popular content, because several attempts must be made to calculate the popularity.

Consequently, this increases the source (cache) utilization. The cache size is limited compared to the giant volume of data being communicated. Owing to the enormous number of replications of similar content, the hit ratio cannot retain its beneficial level to strengthen the caching performance. Another concern is the procedure of changing the cache location based on popular content, which increases the number of eviction-caching operations caused while searching for an empty cache space and for content that has to be replaced.

- How could the content memory consumption be minimized with an improved cache hit ratio?
- How could we enhance the caching mechanism by selecting the centrality position by reducing the stretch ratio?

To answer these questions, a new ICN-based caching strategy is proposed that has the ability to reduce memory consumption with a high cache hit ratio and short stretch for subsequent interests. In addition, it has the ability to minimize content eviction operations.

## 4. Proposed Distributed Caching Strategy (DCS)

In previous studies, it was observed that the ideal structure of the network could affect the overall performance of the system. Cache management is an optimal feature of content centrism, and many researchers have focused on diverse methods of managing disseminated content over networks. Recently, several content caching mechanisms have been developed to increase the efficiency of in-network caching by distributing the transmitted content according to the diverse nature of caching approaches. However, in existing caching mechanisms, several problems related to multiple replications of homogeneous content persist, thereby increasing memory wastage. Content caching mechanisms must implement the optimal objectives to actualize the basic concept of the NDN cache and overcome issues in the data dissemination process which are faced by the aforementioned caching mechanisms [28]. Consequently, in this study, a new, flexible mechanism for content caching has been designed to improve the overall caching performance [29]. The distributed caching strategy works on the popularities of contents. Popularity-based caching strategies are more efficient in terms of improving content dissemination, because these strategies only cache the popular content that can fulfill the requirements of large numbers of consumers, as compared to offensive content. Therefore, the level of popularity of a given piece of content has a significant influence on the caching performance. Mostly, consumers are interested in downloading popular content, and it is a substantial undertaking to cache popular content at the central position. The reason for this is that most incoming interests will be forwarded through the central location. Therefore, if a popular piece of content is cached at the central location, the communication distance will be decreased because all the interests traversing a central position will be accomplished there. Moreover, the central position may also be used to reduce the overall bandwidth consumption. Thus, in this strategy, it becomes more important to cache popular content at centrality positions. This caching strategy is divided into three sections, as shown below:

**Case 1**

**Figure 5:**
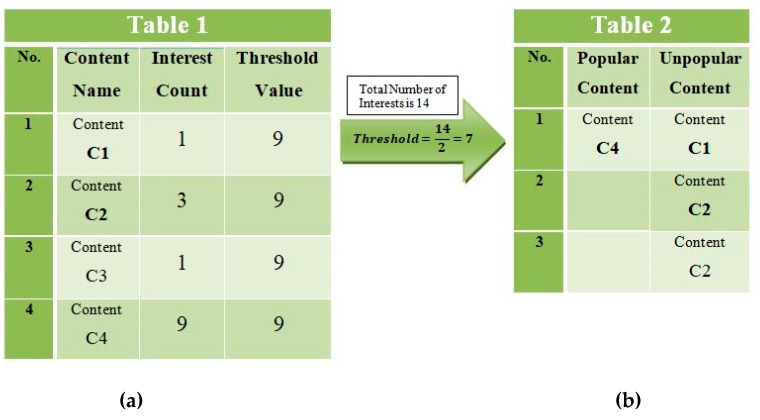
Selection of Popular Content.

The selection of popular content in this strategy is made by taking the sum of the received interests for a specific content name. In the DCS caching strategy, each node is associated with a distinctive statistic table in which information about content name, interest count, and a threshold value is stored. Whenever user interest for particular content occurs, the interest count for a specific piece of content name is incremented with the number of received interests to calculate the popularity of that content. The threshold is a value that is specified to measure the popularity of the content. As a result, if the content receives a number of interests which is equal to the threshold value, it is recommended for classification as “popular”. In earlier popularity-based caching strategies, the threshold is used as statically defined by the strategy algorithm, as described in MPC. However, DCS represents the dynamic threshold to calculate the popularity of a given piece of content. According to DCS, the threshold will be equal to half the total number of received interests for all the contents at a router. Algorithm 1 illustrates the mechanism of selecting popular content. According to the proposed algorithm, if the number of received interests for a particular piece of content is greater than half the total number of interests for all the pieces of content, that content is recommended for classification as “popular”; otherwise, it is ignored. Figure 5 illustrates the mechanism for measuring content popularity. Suppose that 14 searches are generated for Contents C1, C2, C3, and C4, as shown in Figure 5a. According to DCS, Content C4 recommended for classification as “popular” because it has surpassed the threshold value as shown in Figure 5b. Hence, Content C4 is recommended for caching at the intermediate routers along the data delivery path between the user and the provider. Therefore, the first caching operation for popular content will be performed at the closeness centrality router, and secondly, a copy of these contents will also be cached at the edge nodes.


**Algorithm 1: Selection of Popular Content**
1  def Get_Popular_Content() 2     interestRequests=[] 3     interestCount={} 4     popularContents={} 5     unpopularContents={} 6     for interest in interestRequests: 7         if interestCount.__contains__(interest): 8             interestCount[interest]+=1 9         else 10  interestCount.Add(interest,1) 11     threshold=len(interestCount)/2 12    for interest in interestCount.keys(): 13         if interestCount[interest] > threshold: 14             popularContents.Add(interest, interestCount[interest]) 15         else 16             unpopularContents.Add(interest, interestCount[interest]) 17  return popularContents, unpopularContents

**Case 2**

**Figure 6:**
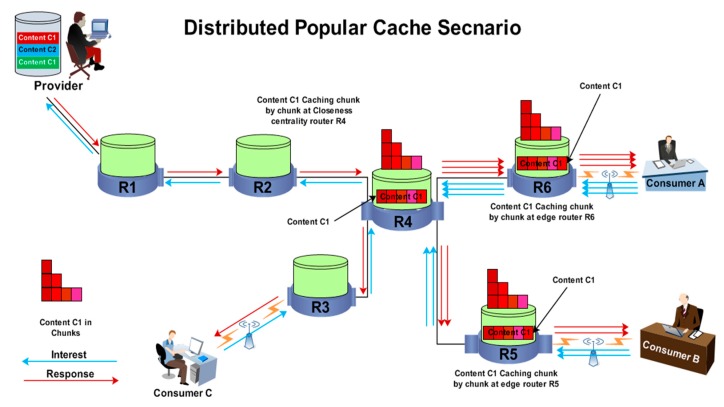
Distributed Caching Strategy.

Popular content cannot be cached in the same way as has been already implemented. The selected contents will be cached in chunk form to reduce the usage of memory and congestion. The reason for this is that the betweenness centrality router is associated with a large number of other routers, which increases the congestion in data dissemination because all interests and contents need to be forwarded through the betweenness centrality router. Therefore, the centrality router has fewer chances to accommodate all popular content at the same time. Thus, the new model increases the ability to cache the maximum quantity of popular content. In DCS, when a content is selected as popular, it is recommended for caching at the closeness centrality router in chunks, as shown in Figure 6 (Distributed Caching Strategy).

Moreover, in terms of chunks, the cache will be used efficiently because its availability will increase to accommodate more content in chunk form. When content is deemed to be popular, it cannot be forwarded to the centrality router until it receives more interest; in response to first interest, only one chunk will be delivered to the closeness centrality and edge router. For subsequent reactions, fragments are multiplied to be forwarded for caching at the closeness centrality router and the edge router. This process will remain functional until the content transfers in its entirety to the centrality router. In this way, if the content was popular, but after being popular, it does not receive any interest, then it will not be cached at the centrality router, and the cache of the centrality router will remain unallocated to accommodate subsequent, more popular contents. In this way, DCS resolves the problem of centrality position and uses the cache in an inefficient manner.

**Case 3**

If a piece of content is deemed to be popular, it will also be forwarded to the edge router at the same time for caching at the closeness centrality router, as shown in Figure 6 (Distributed Caching Strategy). In this way, the path stretch between the consumer and the provider will be reduced for subsequent interests.

Moreover, this will minimize the content retrieval latency for subsequent interests, and reduce the path link congestion by caching the content at edge routers. Therefore, all the following interests will be satisfied with edge routers. If the content is not found at the edge router, then the interest will be satisfied from the closeness centrality router. Moreover, the closeness centrality router is selected for the caching of popular content, because most intents will be accomplished from the centrality router, thereby saving bandwidth consumption with a short stretch path.

For content eviction, the Least Recent (LRU) policy is used to make room for incoming content. The present study proposes a new, ICN-based caching strategy to improve content retrieval latency by reducing the path length between consumers and the provider. Moreover, it reduces the communication path, and network congestion enhances the bandwidth consumption within the limited cache capacity of the network routers.

Figure 6 illustrates the content the caching mechanism in DCS. In the given scenario, Consumers A and B send multiple interests to retrieve Content C1 from the provider. After a while, content C1 becomes popular, because it has received the maximum number of interests that are required to make content popular. Therefore, content C1 is forwarded for caching at closeness centrality router R5. Moreover, the popular content also caches at edge routers R5 and R6. Hence, subsequent interests from Consumers A and B will be satisfied with edge routers R5 and R6. Consequently, Consumer C can download Content C1 from the closeness centrality router.

## 5. Performance Evaluation

For the evaluation of the proposed caching strategy, a simulation platform is used, in which the SocialCCNsim simulator is selected to evaluate the caching performance. The SocialCCNsim [30] simulator was designed to measure caching performance because, in this simulator, all the network routers are associated with cache storage. Cesar Bernardini [31] developed SocialCCNSim based on SONETOR [32], which is a set of utilities that generates synthetic social network traces. These social network traces represent the interactions of users in a social network or a regular client-server fashion. Any caching strategy can be implemented in SocialCCNSim because it was developed especially for ICN-based caching strategies. Two ISP-level topologies were selected to perform a fair evaluation, i.e., Abilene and GEANT. In the final stage, the DCS evaluation was done using simulations, where the chosen parameters were cache size, catalog size, network topology, Zipf probability model, and simulation time. In our simulations, the Zipf probability distribution is used as the popularity model with the α parameter varying between 0.88 and 1.2; the cache size (which specifies the available space in every node for temporally storing content objects) ranges from 100 to 1000 elements (1 GB to 10 GB); and the catalog (which represents the total number of contents in a network) is 107 lements. The performance of the proposed caching strategy is evaluated in terms of memory consumption and the stretch ratio [31].

Moreover, performance is also comparatively evaluated in terms of network contention to measure the cache hit ratio. The proposed caching strategy is compared to ICN centrality-based caching strategies in which FlexPop, CC, and CCS are included. Moreover, categories of contents (User-Generated Content and Video on Demand) are selected with different cache sizes, such as 1 GB to 10 GB. The x-axis of simulation graphs is divided into ten equal parts, in which each part shows the capacities of the cache storage (e.g., from 1 GB to 10 GB). Accordingly, 100 elements show 1 GB and 1000 items 10 GB of cache size. Table 1 shows the simulation parameters. The proposed strategy is evaluated in terms of checking the performance of the most applicable metrics, i.e., memory consumption, path stretch ratio, and cache hit ratio [33].

### 5.1. Memory Consumption

Memory consumption shows the amount of transmitted content that can be cached while downloading the data path for a particular time interval [34]. Consumers can download the contents from multiple routers. In ICN, memory consumption can be clarified as the term of capacity, which shows the volume used by interest and data contents. It can be calculated using the following equation:

(1) $$Memory\;Consumption=\frac{U_{m}}{T_{m}}\times 100$$

where $U_{m}$ shows the memory that is utilized by the cached content and $T_{m}$ presents the total memory (cache storage) of the router along the data delivery path.

**Figure 7:**
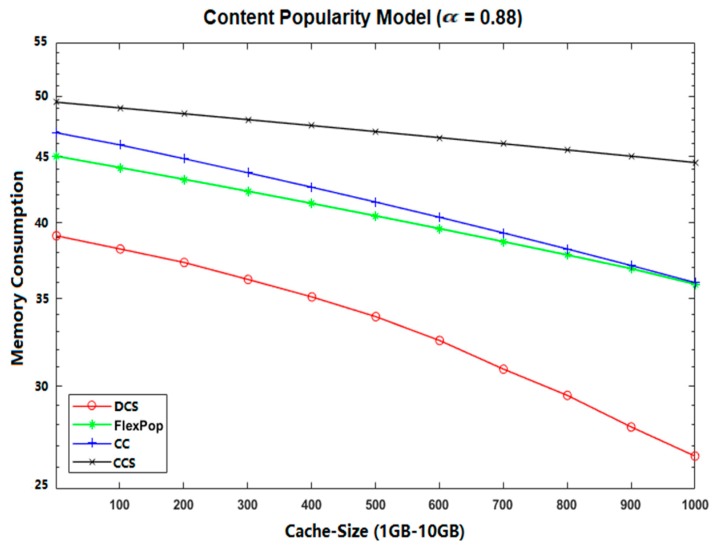
Memory Consumption on Abilene topology with UGC.

**Figure 8:**
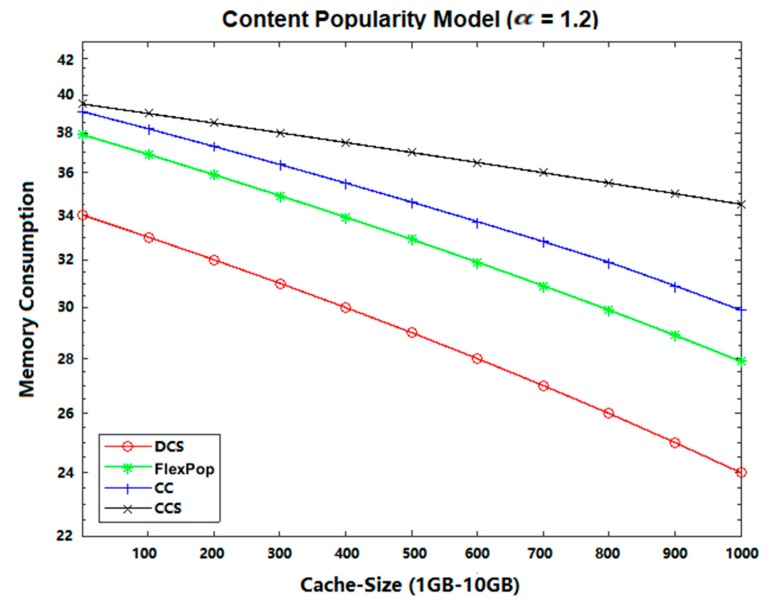
Memory Consumption on GEANT Topology with VoD.

The DCS performs better than CCS, CC, and FlexPop in terms of memory consumption because it provides the ability of chunk level caching of content, thereby decreasing the usage of memory and congestion in path links. Moreover, it delivers the most popular content near consumers, reducing data traffic and allowing contents to move freely across the network. FlexPop and CC deliver poor performance in terms of memory consumption because of their caching of popular content only at a centrality router, a process that increases the traffic congestion within the limited cache capacity. The CCS caches all the content at the betweenness centrality position without considering the content’s popularity, thereby maximizing memory consumption. Figure 7 and Figure 8 show the simulation results on memory consumption using two different topologies (Abilene and GEANT). From these figures, it can be seen that the proposed DCS caching strategy performs much better than FlexPop, CC, and CCS. Thus, we can conclude that DCS is better at enhancing the overall performance of ICN caching in terms of achieving efficient memory consumption.

### 5.2. Stretch

The distance travelled by an interest for a publisher (content provider) is considered as stretch [35,36]. It can be measured using the following equation:

(2) $$\mathrm{Stretch}=\frac{\sum_{i=1}^{I}Hop-traveled}{\sum_{i=1}^{I}Total-Hop}$$

where $\overset{I}{\underset{i=1}{\sum }}Hop-traveled$ represents the number of hops traveled by an interest from the end-user to the content provider. $\overset{\left|I\right|}{\underset{i=1}{\sum }}Total-Hop$ shows the total number of hops from the user to the content provider, and *I* represent the total number of received interests for a given piece of content.

As the cache capacity is small compared to the disseminating content, less content can be accommodated within the centrality routers. Besides, CCS caches all the content without taking their popularity into account; thus, the most popular contents have fewer chances to be cached at the betweenness centrality position due to the unavailability of a popularity module. Hence, overall performance is reduced in terms of a stretch, because all the interests for the most popular contents need to be forwarded to the remote provider, thereby increasing the path length between the consumer and the provider.

The path length is increased for each interest and response. At the same time, the CC and FlexPop cache provide the ability to accommodate popular contents at intermediate locations for a specific time, that can decrease the path length between consumers and providers. The reason for this is that most interests are satisfied with the centrality positions. However, these strategies provide the ability to store popular contents, but due to the limited capacity of the cache at the betweenness centrality router, CC and FlexPop cannot achieve better results in terms of stretch, because both strategies are used to cache less popular contents due to their small thresholds. On the other hand, DCS caches content in a chunk format, increasing the possibility of accommodating more contents.

**Figure 9:**
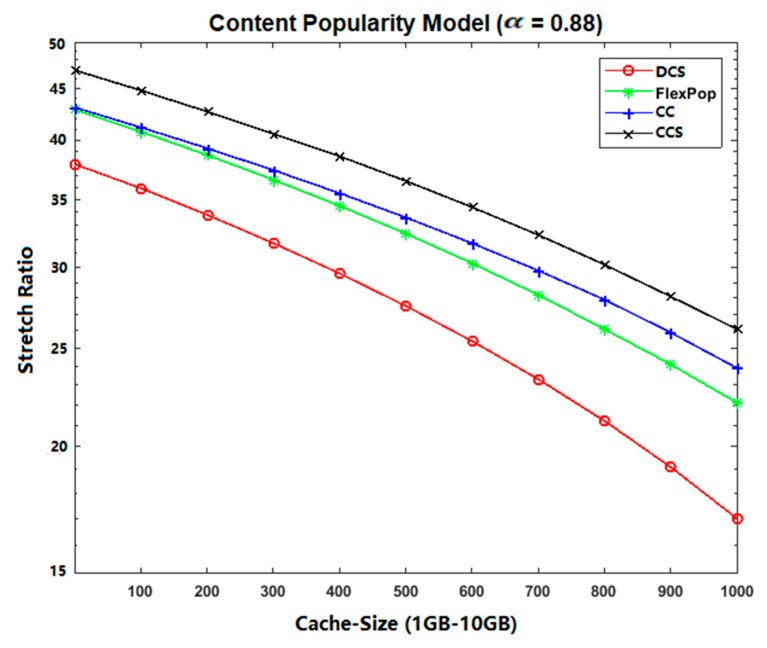
Stretch Ratio on Abilene Topology with UGC.

**Figure 10:**
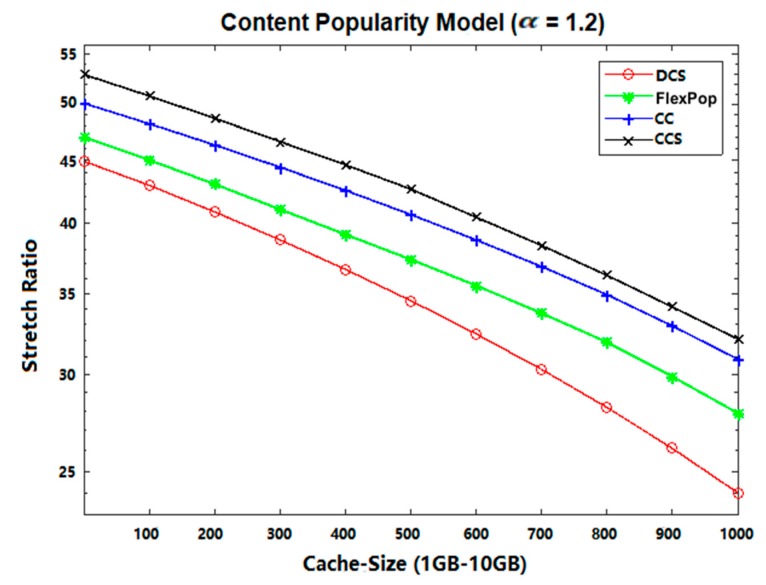
Stretch Ratio on GEANT Topology with VoD.

Therefore, most incoming interests are satisfied with the centrality location. Moreover, the DCS achieves better results in terms of reducing the path stretch because it provides the ability to store content near consumers. Furthermore, it spreads out the cache ability to store chunk level caching of popular content that is used to increase the space available for new popular content. Moreover, DCS caches popular content at edge routers, thereby reducing the path stretch between consumers and providers; therefore, the proposed caching strategy delivers much better results in terms of reducing the overall stretch ratio. From Figure 9 and Figure 10, results indicating that DCS performs better than CCS, CC, and FlexPop are clearly shown.

### 5.3. Cache Hit Ratio

Cache Hit Ratio refers to the quantity of the current content hits as interests are sent [37,38,39] by the consumer to the provider. It can be measured as using the following equation:

(3) $$Cache\;Hit\;Ratio=\frac{\sum_{n=1}^{N}hit_{i}}{\sum_{n=1}^{N}(hit_{i}+miss_{i})}$$

**Figure 11:**
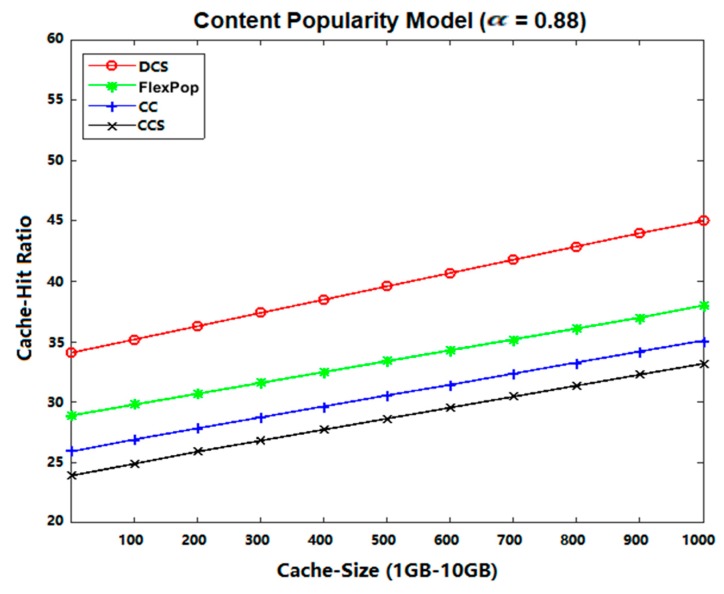
Cache Hit Ratio on Abilene Topology with UGC.

**Figure 12:**
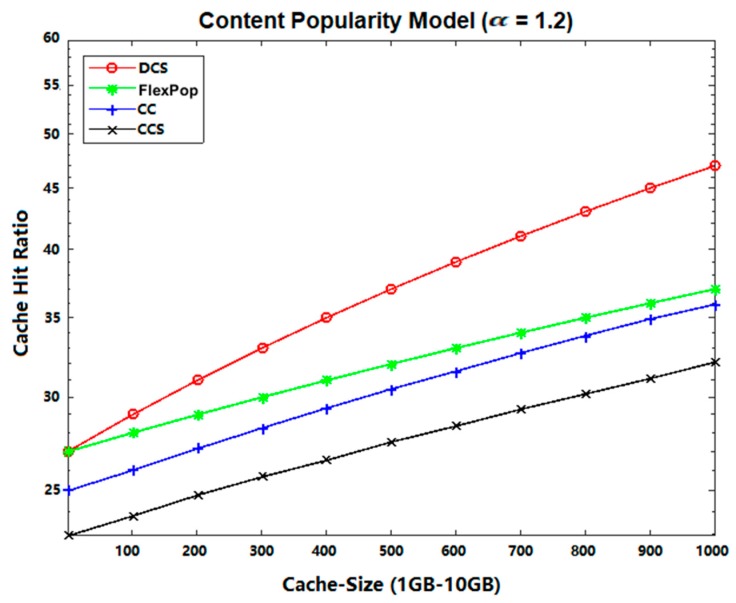
Cache Hit Ratio on GEANT Topology with VoD.

Figure 11 and Figure 12 show the effects of the cache hit ratio on the Abilene and GEANT topologies using different content popularity models. Among the given figures, the DCS caching strategy performed better in terms of a cache hit ratio with both content topologies, because DCS tries to improve the cache allocation of popular contents. Moreover, DCS caches the most popular content at the edge router and closeness centrality routers. Therefore, subsequent interests are satisfied from edge routers, rather than from the remote router.

If an interest cannot be served by the edge router, it is satisfied with the closeness centrality router. Meanwhile, the CCS approach does not define any criteria by which to handle popular content when the cache of the centrality router is full. Therefore, all interests needed to be forwarded to the main data source (or remote router), which increases the path length and decreases the cache hit ratio. In comparison to the CCS approach, the CC and FlexPop approaches performed better. However, both strategies produce a low hit ratio, because fewer contents are accommodated between the centrality routers. On the other hand, DCS caches the content in chunks to increase the availability of storage space at the centrality router. Consequently, we conclude that the proposed DCS strategy performed much better by caching content close to consumers at the network edge.

### 5.4. Eviction Ratio

Content eviction is also one of the significant metrics by which to measure the performance of the caching-based ICN architecture. It can be defined as when the cache of a network node becomes saturated and there is a need to delete some content to accommodate the newly-arriving content. It can be calculated using the following equation:

(4) $$Eviction\;Ratio=\frac{evicted\;content}{total\;content}$$

**Figure 13:**
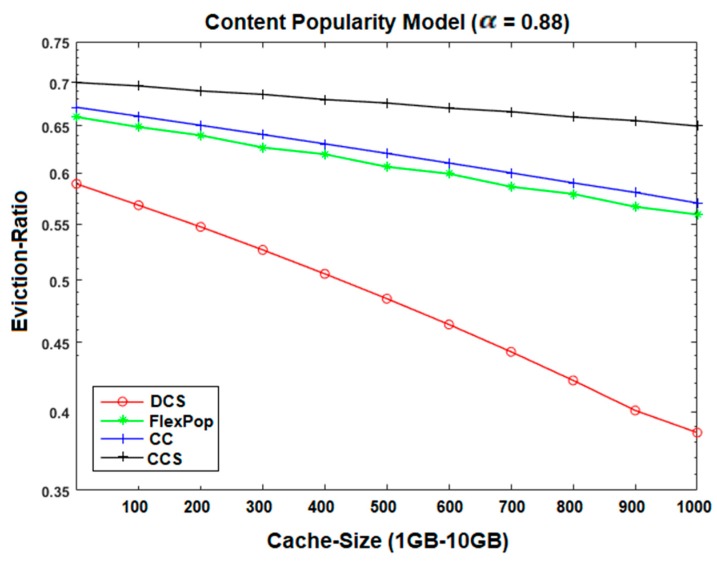
Content Eviction Ratio on Abilene Topology with UGC.

**Figure 14:**
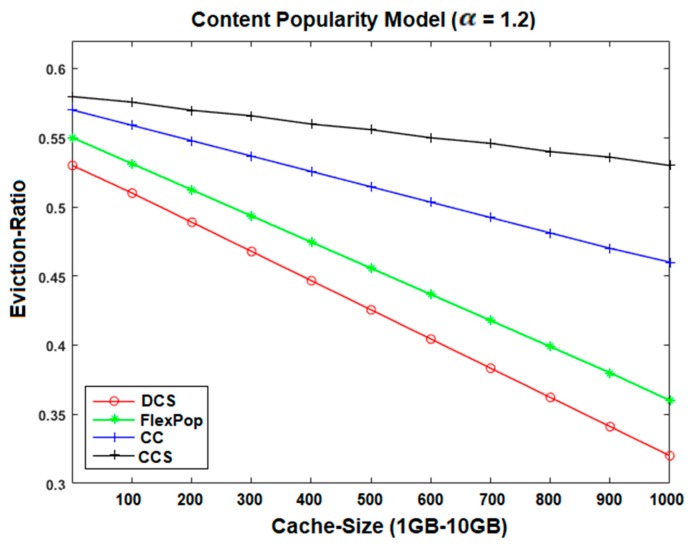
Content Eviction Ratio on GEANT Topology with VoD.

The last number of content evictions disturbs the network throughput and reduces the cache hit and stretch ratios. The reason for this is that all the incoming interests must be forwarded to the distant source to download the appropriate content due to an excessive number of evictions of popular content. Figure 13 and Figure 14 illustrate the outcomes generated by comparisons of centrality-based caching strategies. In the given figure, we can see that the CCS shows a high content eviction ratio, because CCS generally caches all the contents without considering their popularity, and thus, all arriving interests must be forwarded to the remote provider.

CC and FlexPop seem to show better performance in terms of the content eviction ratio, because both strategies are used to cache popular content at centrality routers. However, due to small and static thresholds, these caching strategies cache the least popular contents as well, causing a high number of content evictions. On the other hand, the proposed DCS caching strategy performed better in terms of reducing content eviction ratio as compared to CC, CCS, and FlexPop caching strategy. The reason is that the DCS distributes and caches the content in chunks format that increases the overall cache storage to accommodate the new contents. Besides, it uses to cache on the most popular content at centrality routers that increase the availability of free cache to provide popular content. Moreover, DCS caches the least popular content at the edge routers, and therefore, the subsequent interests are accomplished from the nearest routers. Thus, DCS minimizes the content eviction ratio by caching the least popular content at edge routers and the most popular content at centrality routers.

## 6. Conclusions

The new search and big data technology will deliver a massive amount of data that will be difficult to handle by using the current IP-based internet architecture. The reason is that the existing internet architecture supports the addresses based data communication which will be insufficient to fulfill the future requirements related to location-based data transmission. Similarly, the information dissemination in the current VSNs also depend on physical location in which similar data is transmitted several times across the network. This data replication has led to several problems in which resource consumption (memory), stretch, and communication latency due to the lake of data availability, are the most crucial issues. ICN provides an enhanced version of the internet that can provide the ability to resolve such issues efficiently. ICN is a new internet paradigm that supports innovative communication systems with location-independent data dissemination. ICN with VSN can handle the massive amount of data generated from heterogeneous mobile sensors in surrounding smart environments. Therefore, new ICN paradigms are emerging as a new technology to enhance communication processes for VSNs. Moreover, it can reduce the number of difficulties in the current internet paradigm; it provides edge routers in a VSN that can store the disseminated content for a specific time, while taking the required memory consumption, stretch ratio, and hit ratio into account. To improve the performance of content dissemination in an ICN-based cache of vehicles, a new caching strategy is proposed to provide less memory consumption, a low stretch ratio, a low content eviction ratio, and a high cache hit ratio by caching the most desired content close to consumers.

## 7. Future Directions

The requirements for enhancing the VNS infrastructure are rapidly expanding, because content generation and dissemination require more volume than the currently network capacities. Consumers are interested in data-needed contents, rather than data source locations. The reason for this is that the existing internet architecture supports location-based content routing, which increases the amount of network traffic; similar contents are transferred multiple times to satisfy consumers’ needs. This redundant content routing process generates several problems, e.g., congestion, high bandwidth usage, and resource consumption (power and energy). Consequently, these critical problems have to be resolved by using an efficient, scalable, and reliable (secure) architecture for the internet [40,41]. The VNS is a new promising architecture that integrates several technologies and communication developments for the mobile internet. It provides several benefits, using identification and tracking technologies for wireless networks.

The most significant feature of the ICN is a cache that is used to store popular contents in order to serve user requests. In vehicular networks, vehicles can obtain their required contents from neighboring vehicles in short time with a small stretch [42]. Therefore, there is no need to forward incoming interests to remote providers. A large number of interests are generated for the same content from several vehicles, and vehicles are unable to retrieve the required content directly from the base station within partial coverage situations [43]. In this situation, the proposed caching strategy will significantly decrease the burden on the original provider, and will provide efficient data dissemination services [44]. Moreover, it offers distributed intelligence for smart objects (vehicles) [43]. VNS technology delivers benefits to mobile, interconnected nodes (vehicles), such as informatics, telecommunication, social science, and electronics. However, VSN still faces several complications, owing in no small part to the amount of data that is produced from heterogeneous devices (vehicles). Numerous diverse sensors are required in VSN, thereby increasing power and resource consumption [2]. Furthermore, VSN devices transmit a tremendous amount of content that is difficult to manage using the current IP-based internet architecture. In these situations, DCS introduces an enhanced scheme for data transmission across the internet, and it can overcome the current challenges of the IP-based internet and VSN [1].

The vast number of smart devices generates a significant amount of content that can be managed efficiently by the implementation of the DCS caching strategy. DCS provides content to network nodes, and all the nodes can store the disseminated contents during their transmission near the consumers at the intermediate nodes. Consequently, they can fulfill the requirements of subsequent interests in a shorter period compared to the retrieval of content from remote content providers. Moreover, the DCS caching strategy can reduce the power and resource consumption by caching content near users in chunk form. Thus, if a source node in the VSN is unreachable, consumers can still retrieve their desired content from any other caching node. The integration of DCS within the VSN can increase the reliability of the VSN architecture by deploying content near end users [45].

## Figures and Tables

**Table 1:** Simulation Parameters.

Parameter	Description
Simulation time	24 h
Topologies	Abilene and GEANT
Content Size	10 MB each
Catalog Size	10^7^ elements
Cache Size	100 to 1000 elements
α Parameter	0.88 and 1.2
Content Categories	UGC and VoD
Simulator	SocialCCNSim
Social Network Topology	Facebook
Traffic Source	SONETOR
Metrics	Memory Consumption, Cache Hit Ratio, Stretch Ratio, and Content Eviction Ratio.
